# Pancreatic Inflammatory Pseudotumor-Like Follicular Dendritic Cell Tumor

**DOI:** 10.1155/2019/2648123

**Published:** 2019-12-05

**Authors:** Madison Mograbi, Michael S. Stump, David T. Luyimbazi, Mohammad H. Shakhatreh, Douglas J. Grider

**Affiliations:** ^1^Eastern Virginia Medical School, Norfolk, VA, USA; ^2^Department of Basic Science Education, Virginia Tech Carilion School of Medicine, Roanoke, VA, USA; ^3^Dominion Pathology Associates, Roanoke, VA, USA; ^4^Department of Surgery, Carilion Clinic and Virginia Tech Carilion School of Medicine, Roanoke, VA, USA; ^5^Gastroenterology Division, Department of Medicine, Carilion Clinic and Virginia Tech Carilion School of Medicine, Roanoke, VA, USA

## Abstract

Follicular dendritic cell sarcoma (FDCS) is a rare and underdiagnosed malignant neoplasm which characteristically presents as a solitary, slow-growing mass with no discrete symptoms. Histologically, lymphocytes and spindle cells featuring large nucleoli in a whorled pattern are usually seen. FDCS is classically found in cervical and axillary lymph nodes, with occasional involvement of extranodal sites. Inflammatory pseudotumor-like follicular dendritic cell tumor (IPT-like FDCT) is an uncommon subcategory of this neoplasm, demonstratively linked to the Epstein-Barr virus (EBV). This neoplasm can present similarly to FDCS, but systemic symptoms may be seen. Although, often found in the spleen and occasionally the liver, IPT-like FDCT has not previously been described within the pancreas. Presented, is an IPT-like FDCT of the pancreas and spleen of a 70 years old woman. Histologic features include variably sized geographic suppurative granulomas with chronic inflammatory cells and an atypical spindle cell proliferation with prominent nucleoli. Positivity for CD45 and CD68 in the larger spindled cells points to an inflammatory pseudotumor subtype and co-expression of CD21, CD23, and CD35 were indicative of follicular dendritic differentiation. The pseudotumor additionally demonstrated EBV-encoded RNA (EBER) positivity typical of IPT-like FDCT. Differentiation between inflammatory pseudotumor (IPT) and inflammatory myofibroblastic tumor (IMT) is additionally discussed.

## 1. Introduction

Follicular dendritic cell sarcoma (FDCS) is extremely rare and underdiagnosed, occurring primarily in lymph tissue and uncommonly in extranodal sites such as the spleen, liver, and tonsils [1]. Metastatic disease has also occurred, especially when the site of origination is the liver, affecting distant sites such as the lung and gastrointestinal system [2]. Inflammatory pseudotumor-like follicular dendritic cell tumor (IPT-like FDCT) is an uncommon subset of this neoplasm whose etiology is unknown; only about 38 cases of reported documentation of this tumor are available, none of which involve the pancreas. This neoplasm commonly presents as a unifocal, slow-growing, painless mass similar to FDCS, but behaves differently and is histologically unique. IPT-like FDCT can present with systemic symptoms, has a female predominance (2-3 : 1), and a median age of 57 years [3]. Histologically, IPT-like FDCT is similar to inflammatory pseudotumor (IPT) and contains loose spindle cells, focal sclerosis, and mixed inflammatory infiltrates, and up to ~90% of known splenic IPT-like FDCT cases show Epstein-Barr virus (EBV) positivity [3]. This neoplasm appears indolent and prognosis is favorable. Due to the gross and histological nature and complexity of IPT-like FDCT, misdiagnoses are possible, resulting in the neoplasm likely being underdiagnosed. CT and MR findings are not diagnostic due to the nonspecificity of IPT-like FDCT, and histologically, diagnosis is principally achievable through antigen markers and evidence of EBV infection.

Also within the differential, inflammatory myofibroblastic tumor (IMT) has emerged over the past thirty years as a subcategory of inflammatory pseudotumor. IMT presents as a mass with systemic symptoms including abdominal pain, fever, malaise, and weight loss. These tumors resemble those of IPT-like FDCT, displaying firm or fleshy white cut surfaces and are typically solitary. Histologically, similarities to IPT-like FDCT have been noted. IMT has been used interchangeably with IPT until recent studies, making an understanding of these entities more difficult.

## 2. Case Study

**Figure 1:**
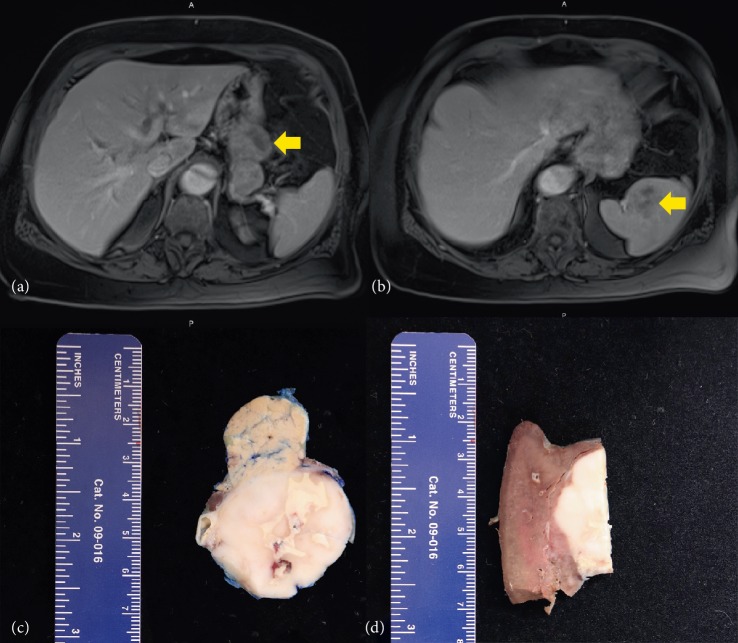
(a and b) showing contrast-enhanced T1 MR images of the pancreatic (a) and splenic (b) lesions, arrows point to the lesions; (c) shows the gross image of the IPT-like FDCT in the pancreas; (d) shows the gross image of the IPT-like FDCT in the spleen.

A seventy-year-old woman with an extensive medical history, including a Stanford B aortic dissection, pituitary adenoma, meningioma, and acoustic neuroma, presented with an incidental solitary, painless mass located in the left upper quadrant. Multiplanar, multisequence magnetic resonance imaging was performed with and without 7 mLs MultiHance contrast to reveal a 7.0 cm anterior–posterior × 4.4 cm, transverse × 4.0 cm craniocaudad, dumbbell-shaped mass in the pancreatic tail containing no clear dividing plane of fat between the mass and the posterior gastric body wall or the superior margin of the pancreas (Figure 1). A second, noncontiguous mass was additionally identified within the splenic hilum. On imaging, both masses appeared to restrict diffusion of the organs (Figures 1(a) and 1(b)). No palpable lymphadenopathy was found. Robotic distal pancreatectomy and splenectomy was indicated to the patient and subsequently performed. Grossly, the portion of distal pancreas measured 8.5 × 5.5 × 3.2 cm and contained a single, well-circumscribed, firm, white nodule, measuring 7.0 × 4.2 × 4.0 cm, the cut surfaces of which displayed irregular yellow foci and gray regions of necrosis (Figure 1(c)). The portion of spleen measured 14.0 × 8.0 × 4.0 cm and contained a solitary mass featuring morphology similar to that of the pancreas, measuring 3.0 × 2.7 × 2.8 cm (Figure 1(d)). Grossly, the suspected diagnosis was consistent with that of a lymphoma.

**Figure 2:**
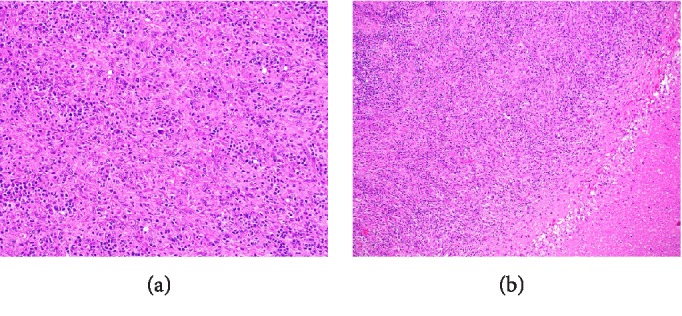
Composite H&E. (a) Shows atypical spindle cell proliferation with intermixed chronic inflammatory cells (200 magnification); (b) shows suppurative granuloma with palisade of histiocytes and overlying atypical spindle cell proliferation (100 magnification).

**Figure 3:**
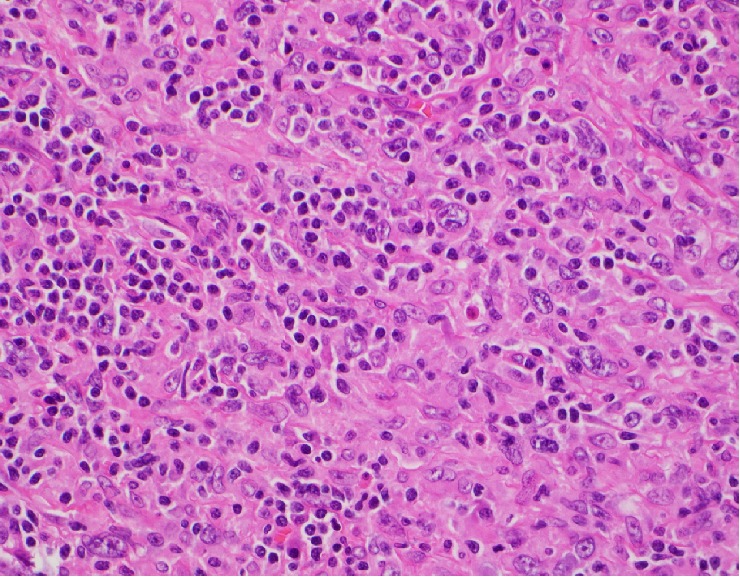
High power H&E of atypical spindle cells with intermixed lymphoplasmacytic inflammation (400 magnification (40X)).

**Figure 4:**
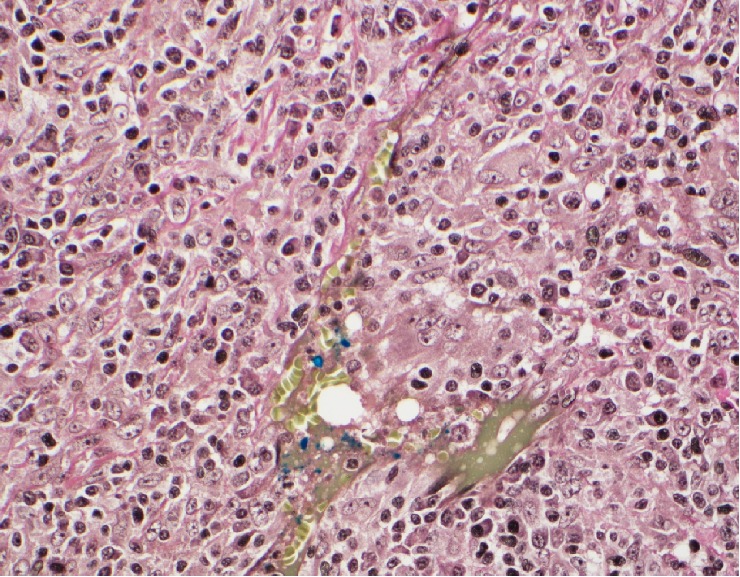
Reticulin stained tissue section showing nonobstructive phlebitis (400 magnification (40X)).

Histologic features of both masses were similar and consistent with the same type of neoplasm. Variably sized geographic noncaseating suppurative granulomas were dispersed uniformly throughout the tumor. These epithelioid granulomas had an associated intermixed lymphoplasmacytic inflammatory infiltrate and a prominent atypical spindle cell proliferation (Figure 2). These cells contained variably sized nuclei, prominent nucleoli, and distinctly irregular nuclear contours (Figure 3). No discrete storiform pattern of fibrosis was noted on either a haematoxylin and eosin or elastic stain; however, obliterative phlebitis was focally noted, as well as phlebitis without obstruction of the lumen (Figure 4). This pattern of behavior has previously been demonstrated in IPT as well as IPT-like FDCT [4–6]. There was no obvious lymphomatous cell population identified and immunohistochemical stains failed to identify an obvious lymphoma.

**Figure 5:**
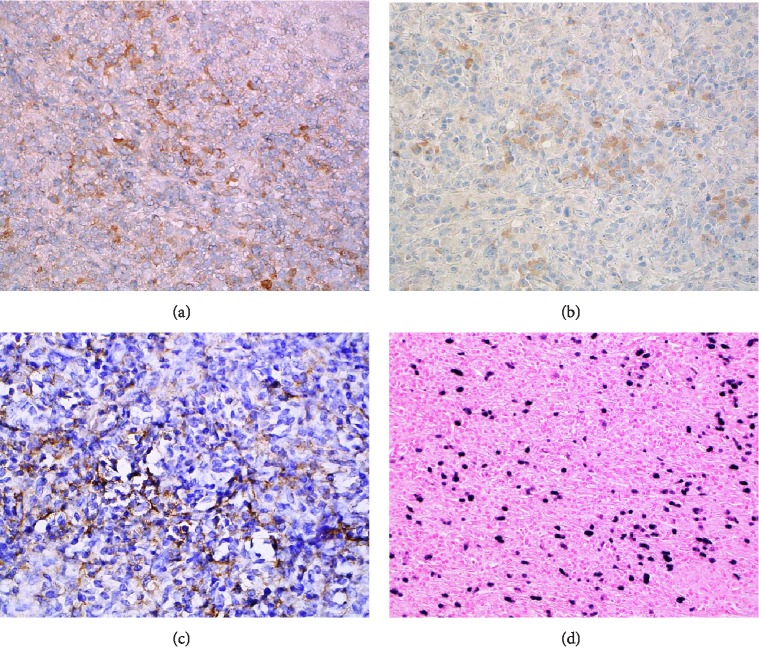
Composite ancillary studies confirming IPT-like FDCT. (a) CD21 immunohistochemical positivity (400 magnification); (b) CD23 immunohistochemical positivity (400 magnification); (c) CD35 immunohistochemical positivity (400 magnification); (d) EBV-encoded (EBER) in situ hybridization positivity (200 magnification).

Chromogenic in situ hybridization (CISH) for EBV-coded RNA (EBER) as well as an extensive panel of immunohistochemical markers were performed with appropriate controls. The tumor cells demonstrated CD68 positivity, supporting an inflammatory pseudotumor. Smooth muscle actin was negative, suggesting against an inflammatory myofibroblastic tumor (IMT). The tumor was negative for ancillary stains for organisms, including AFB, GMS, PAS, and gram-stained sections. To rule out a lymphoma, CD45, CD3, PAX-5, CD15, CD30, and CD20 antigen tests were completed. CD45 marked background lymphoid cells as well as larger spindled cells, which has been previously described in inflammatory pseudotumors [7]. CD3 marked some background T-cells. PAX-5 and CD20 marked minimal aggregates of B-lymphocytes, likely consistent with residual germinal centers. CD15 marked few inflammatory cells. CD30 and EMA were negative. Keratin CAM 5.2 and pankeratin stains were negative, excluding a spindled cell carcinoma. The neoplastic follicular dendritic cells expressed CD21, CD23, and CD35 at least focally and demonstrated abundant cells positive for in situ EBV hybridization, consistent with IPT-like FDCT (Figure 5).

## 3. Discussion

IPT-like FDCT is likely an underdiagnosed neoplasm that has been documented predominantly in the spleen but with occasional cases involving the liver. IPT-like FDCT has the potential to recur or metastasize and is considered by most as a low-grade sarcoma. While some IPT cases have been associated with surgical or traumatic inflammation as well as autoimmune conditions and infection like *T. pallidum*, IPT-like FDCT has only consistently been associated with EBV [8, 9].

Reviewing the literature on the general category of IPT and IMT is challenging because these two terms have been used interchangeably, especially in early publications [10, 11]. However, these entities are now accepted as separate, with IMT being recognized as a clonal disorder while IPT is not. This discrepancy makes evaluating early reports difficult and inconclusive, as the unique subtypes were previously diagnosed together and treated equal. Given these entities' similarities it is not surprising that a possible relationship between IPT/IMT and FDCS was described by Neuhauser et al. [7]: “It has been suggested that IMT (IPT) and FDC tumors may be related, sharing a common myofibroblastic lineage.”

Differentiating IPT-like FDCT from IPT, IMT, and FDCS requires knowledge of the clinical picture as well as use of immunohistochemical stains. IPT-like FDCT characteristically expresses at least one follicular dendritic cell (FDC) marker and shows prominent positivity for EBV on in situ hybridization. The amount of FDC expression is variable with some cases only showing focal expression. This immunophenotype differs from IMT which prominently expresses SMA, is negative for FDC markers, and does not characteristically show EBV positivity. Additionally, a significant subset of IMT cases express ALK, especially in younger patients, although splenic IMT is usually negative. IPT can be differentiated as it also does not express FDC markers and is usually negative for EBV or only minimally positive. Differentiation from the more aggressive FDSC is important and this distinction can be made as FDSC is clinically more aggressive, tends to involve lymph nodes rather than the spleen, and does not typically display EBV positivity or a prominent inflammatory background.

## 4. Summary/Conclusion

The present case explored pancreatic and splenic tumors in a seventy-year old woman, each demonstrating well-circumscribed, firm white cut surfaces. Histologic examination revealed epithelioid noncaseating granulomas associated with a spindle cell proliferation and lymphoplasmacytic inflammatory infiltrate. EBV positivity was prominent and atypical spindle cells demonstrated positivity for CD68 and focal positivity for CD21, CD23, and CD35 while being negative for SMA. Lymphoma was also excluded via CD45, CD3, PAX-5, CD15, CD30, and CD20 negativity as well as the lack of a morphologically identifiable malignant lymphoid cells or Reed Sternberg cells. These findings are consistent with previous studies and lead to our eventual diagnosis of IPT-like FDCT.

IPT-like FDCT is a rare neoplasm with a favorable prognosis. Often seen as a solitary mass with or without symptoms involving the spleen or liver, IPT-like FDCT has not previously been identified in the pancreas. The present case describes the first known instance of this rare subtype involving pancreas and how the conclusion of IPT-like FDCT was reached. For this neoplasm, surgical removal of the mass tends to lead to resolution with only few cases of recurrence and a single case of death due to disseminated intraperitoneal disease [2, 12, 13].
